# Glutamate transmission in the prelimbic cortex and nucleus accumbens shell is involved in ethanol reinforcement and drinking in rats

**DOI:** 10.3389/fnbeh.2026.1745128

**Published:** 2026-02-17

**Authors:** Xiaoying Tan, William J. McBride, Zheng-Ming Ding

**Affiliations:** 1 Department of Anesthesiology and Perioperative Medicine, Penn State University College of Medicine, Hershey, PA, United States; 2 Department of Psychiatry, Indiana University School of Medicine, Indianapolis, IN, United States; 3 Department of Neuroscience and Experimental Therapeutics, Penn State University College of Medicine, Hershey, PA, United States

**Keywords:** ethanol, glutamate, intra-cranial self-administration, microdialysis, nucleus accumbens shell, prelimbic cortex

## Abstract

**Introduction:**

Research has implicated mesocorticolimbic glutamate transmission in alcohol use. The current study focused on glutamate transmission within two key sub-regions, i.e., prelimbic (PL) cortex and nucleus accumbens (NAc) shell, in mediating ethanol reinforcement and drinking in rats.

**Methods:**

Intracranial self-administration (ICSA) was conducted to examine effects of inhibition of local glutamate transmission on ethanol ICSA into these sub-regions. Protein levels were determined with Western blot in both sub-regions of alcohol-preferring P and Wistar rats on key glutamate-related proteins that can regulate extracellular glutamate levels. Quantitative microdialysis was performed to measure basal extracellular glutamate concentrations and clearance in the PL cortex following chronic ethanol drinking. An additional study tested effects of centrally administered LDN-212320, a glutamate transporter 1 (GLT-1) activator, on ethanol drinking.

**Results:**

Co-infusion of the metabotropic glutamate receptor 2/3 (mGluR2/3) agonist LY379268 with ethanol inhibited ethanol ICSA into these sub-regions. Expression of mGluR3, but not GLT-1, was lower in P than Wistar rats in both sub-regions. Ethanol drinking enhanced basal extracellular glutamate concentrations and reduced glutamate clearance. Intra-ventricular microinjection of LDN-212320 decreased ethanol drinking.

**Discussion:**

These results suggest that (a) activation of local glutamate transmission is critical to ethanol reinforcement within the PL cortex and NAc shell, (b) strain difference exists in mGluR3 protein expression between P and Wistar rats, (c) chronic ethanol induces neuro-adaptations characterized by enhanced basal extracellular glutamate transmission, and (d) up-regulation of GLT-1 attenuates ethanol drinking. Taken together, these results further support the importance of glutamate transmission within the PL cortex and NAc shell in mediating ethanol effects.

## Introduction

Alcohol use remains a serious public health concern. The 2022 National Survey on Drug Use and Health reported that 48.7% (137.4 million) of people aged 12 or older in the United States were current alcohol users and 10.5% (29.5 million) of this population had a past year alcohol use disorder (Substance Abuse and Mental Health Services Administration [SAMHSA], 2023). Excessive alcohol use takes a heavy toll on individuals and society in the United States, causing ∼178,000 deaths, ∼4 million years of potential life lost, and over $250 billion economic loss yearly (Centers on Disease Control and Prevention [CDC], 2024). Three medications are currently FDA-approved for treatment of alcohol use disorder, including disulfiram, naltrexone, and acamprosate. Despite their clinical benefits, these medications are only moderately effective and their clinical use is limited, highlighting the great need for better understanding of brain mechanisms underlying alcohol use (Koob and Mason, 2016; Kranzler and Hartwell, 2023).

A substantial body of literature indicates that two key sub-regions within the mesocorticolimbic system, i.e., nucleus accumbens (NAc) shell and prelimbic (PL) cortex, play important roles in the development of alcohol use (Koob and Volkow, 2010). Studies using the intracranial self-administration (ICSA) technique demonstrate that ethanol can be self-infused directly into the NAc shell and PL cortex in rats, suggesting that these regions may be important brain anatomical substrates underlying acute reinforcing effects of ethanol (Engleman et al., 2009, 2020). Studies indicate that ethanol alters neurotransmission within these sub-regions. For examples, acute ethanol increases extracellular dopamine levels in both sub-regions (Ding et al., 2009a,^2011^; Imperato and Di Chiara, 1986; Schier et al., 2013; Weiss et al., 1993). Chronic ethanol use induces neuroadaptations, e.g., in dopamine system, in both sub-regions (Thielen et al., 2004; Trantham-Davidson et al., 2014; Trantham-Davidson et al., 2017). In addition, pharmacological manipulations of neurotransmission within these two sub-regions alter ethanol drinking and self-administration (Ding et al., 2015b; Hodge et al., 1996; Rassnick et al., 1992; Samson and Chappell, 2003).

Glutamate transmission has been implicated in ethanol’s effects and the development of ethanol use. Acute ethanol inhibits glutamate receptor-mediated currents (Lovinger et al., 1989; Steffensen et al., 2000; Woodward, 1999). Systemic administration of ethanol alters extracellular glutamate levels in different brain regions (Ding et al., 2012; Moghaddam and Bolinao, 1994; Piepponen et al., 2002; Selim and Bradberry, 1996). Chronic alcohol use induces a hyper-glutamatergic state characterized, at least, by enhancement of basal extracellular glutamate levels across various brain regions (Dahchour and De Witte, 1999; Ding et al., 2012, 2013; Kapasova and Szumlinski, 2008; Melendez et al., 2005). Furthermore, numerous studies show that glutamate receptor ligands alter ethanol drinking and reinforcement (Gass and Olive, 2008).

However, several questions remain regarding the role of glutamate transmission within the NAc shell and PL cortex in ethanol reinforcement and drinking. For example, our ICSA studies indicate that local reinforcing effects of ethanol within the PL cortex require activation of local dopamine neurotransmission, and those within the NAc shell involve activation of serotonin and GABA neurotransmission (Ding et al., 2015a; Engleman et al., 2020). However, a potential involvement of glutamate transmission in these acute effects of ethanol remains unknown. Our studies indicate that chronic ethanol drinking increases extracellular glutamate levels and reduces glutamate clearance within both VTA and NAc shell (Ding et al., 2012, 2013). But it remains unexplored whether ethanol drinking would induce similar or different effects in the PL cortex. In addition, our study shows that the metabotropic glutamate receptor 2 (mGluR2) is expressed in Wistar rats, but not in alcohol-preferring P rats in both PL cortex and NAc shell (Ding et al., 2017). mGluR2 is mainly a glutamate auto-receptor that regulates glutamate release and extracellular glutamate levels. However, it remains unknown whether strain differences exist in other glutamate-related proteins known to regulate extracellular glutamate levels, e.g., the mGluR3 auto-receptor and the glutamate transporter 1 (GLT-1) which provides the majority of extracellular glutamate clearance.

Given these, the objective of this study was to further characterize the involvement of glutamate transmission within the PL cortex and NAc shell in ethanol reinforcement and drinking. This study investigated (1) effects of activation of local glutamate transmission in the reinforcing effects of ethanol within these sub-regions, (2) protein expression of mGluR3 and GLT1 in both sub-regions, and (3) effects of chronic ethanol drinking on basal extracellular glutamate levels and clearance in the PL cortex. Furthermore, a series studies have shown that several antibiotics may reduce ethanol drinking in rats through enhancement of GLT1 expression in the mesocorticolimbic system (Alhaddad et al., 2014; Goodwani et al., 2015; Qrunfleh et al., 2013; Sari et al., 2011). Therefore, this study also examined effects of LDN-212320, a translational activator of GLT1 (Colton et al., 2010; Xing et al., 2011), on ethanol drinking following central administration.

## Materials and methods

### Animals

Adult Wistar rats (Envigo, Inc., Indianapolis, IN) and alcohol-preferring P rats (Indiana University) were used in the present study. Each experiment involved only one sex of animals. ICSA experiments included mainly female rats as performed in our previous studies because female rats tend to maintain their head size better for consistent placements of microinjector in the target region during prolonged ICSA experiments (Ding et al., 2009b,2015a, b; Engleman et al., 2009, 2020). Rats were kept on a reversed 12 h light-dark cycle with food and water available *ad libitum*. Rats were housed in pair upon arrival and were housed individually after surgery and during ethanol drinking. Samples sizes were determined mainly based on our previous studies (Ding et al., 2013, 2015a,^2017^; Engleman et al., 2020). Protocols used were approved by the Institutional Animal Care and Use Committee at Indiana University School of Medicine for protocol #11313 and the Pennsylvania State University College of Medicine for protocol #201900952. All experiments were performed in accordance with the principles outlined in the Guide for the Care and Use of Laboratory Animals (National Research Council, 2011).

### Chemical agents

Sodium chloride, potassium chloride, magnesium chloride, potassium phosphate monobasic, sodium phosphate, magnesium phosphate, sodium bicarbonate, calcium chloride, d-glucose, methanol, glutamate, dimethyl sulfoxide (DMSO), polyethylene glycol 400, TWEEN^®^ 80, and (2-hydroxypropyl)-β-cyclodextrin were purchased from Sigma-Aldrich (St. Louis, MO). O-phthalaldehyde was obtained from Pickering Laboratory, Inc., (Mountain View, CA). LY379268 and LDN-212320 were purchased from Tocris (Minneapolis, MN). Ethanol (190 proof) was obtained from McCormick Distilling, Weston, MO. LDN-212320 was dissolved in a vehicle containing 2.5% DMSO, 1% polyethylene glycol 400, 0.2% tween 80, 10% hydroxypropyl-b-cyclodextrin in saline.

### Stereotaxic surgery

Intracranial cannulation surgery followed general procedures previously described (Ding et al., 2015a; Engleman et al., 2020). Briefly, rats received stereotaxic surgery under anesthesia with inhalation of 2%–3% isoflurane. Guide cannulae were unilaterally implanted aimed at the target brain regions. Cannulae of 22-gauge [Inner diameter (I.D.) × Outer diameter (O.D.) = 0.39 mm × 0.71 mm; Plastics One Inc., Roanoke, VA) were used for ICSA and microinjection studies, and cannulae of 18 gauge (I.D. × O.D. = 0.82 mm × 1.27 mm; Plastics One, Inc., Roanoke, VA, United States) were used for microdialysis studies. Coordinates of these brain regions were determined according to the Rat Brain in Stereotaxic Coordinates (Paxinos and Watson, 1998), including PL cortex (AP +3.0 mm, ML +0.7 mm, DV −3.0 mm), NAc shell (AP +1.7 mm, ML +2.4 mm, DV −7.0 mm), or lateral ventricle (AP +1.3 mm, ML −0.9 mm, DV −3.0 mm). Stylets were inserted into guide cannulae after surgery.

### ICSA

Standard procedures were followed as previous described (Engleman et al., 2009, 2020). Wistar rats were selected in this study because our previous ICSA studies in these two regions mainly used Wistar rats (Engleman et al., 2009, 2020). During ICSA tests, ethanol-naïve rats were placed into operant chambers equipped with two levers, one active and the other inactive, counterbalanced among rats. The active lever was connected to an A-M Model 2100 isolated pulse stimulator (A-M systems, Inc., Carlsborg, WA) that was controlled by an operant conditioning control system (Coulbourn Instruments, Allentown, Pennsylvania, United States). The pulse stimulator was connected to two electrodes that were immersed and secured in a solution-filled cylinder container. The container was equipped with a 28-gauge injector cannula (I.D. × O.D. = 0.18 mm × 0.36 mm; Plastics One Inc., Roanoke, VA), and was inserted into and screwed on the guide cannula with 1 mm extension into the target brain region. A fixed-ratio 1 schedule was used. Each response on the active lever activated the pulse stimulator and generated electric current between the two electrodes, resulting in an infusion of 100 nl solution into the target region over 5 s. Each infusion was followed by a 5 s timeout period. During both the infusion and timeout periods, responses on active lever were recorded but did not elicit additional infusions. Responses on inactive lever were recorded with no programed consequences.

Rats started with ICSA of 150 mg% ethanol dissolved in artificial cerebrospinal fluid (aCSF: 120 mM NaCl, 4.8 mM KCl, 1.2 mM KH_2_PO_4_, 1.2 mM MgSO_4_, 25 mM NaHCO_3_, 2.5 mM CaCl_2_, 10 mM d-glucose, pH 7.2–7.4) into either PL cortex (*n* = 5–6/group) or NAc shell (*n* = 6–8/group) for the first four sessions. These were designated as acquisition sessions. Ethanol at 150 mg% has been shown to be one optimal concentration in eliciting robust ICSA in the NAc shell and PL cortex (Engleman et al., 2009, 2020). During sessions five and six, rats received ICSA of 150 mg% ethanol in combination with LY379268 (0, 100, 330, or 1,000 μM). These were designated as co-infusion sessions. During session seven, rats returned to ICSA of only 150 mg% ethanol, which was designated as the re-acquisition session. This seven-session procedure has been routinely performed in our group for investigating receptor mechanisms involved in ethanol ICSA (Ding et al., 2009b,2015a, b; Engleman et al., 2009, 2020). Sessions were 4 h in duration and were conducted every 48–72 h. LY379268 is a potent agonist of mGluR2/3 receptors, and activation of these receptors reduces presynaptic glutamate release and extracellular synaptic glutamate levels (Imre, 2007). Therefore, this study examined the involvement of local extracellular glutamate transmission in the reinforcing effects of ethanol within these brain regions. Three rats were excluded from sample size count and analysis due to placements outside of NAc shell.

### Western blot

Protein levels were determined between alcohol-naïve Wistar and P rats with Western blot as previously described (Ding et al., 2013, 2017). Briefly, brains were harvested from rats (*n* = 7/group) and were sliced with a cryostat into 300 μm sections. Brain tissue was micro-punched from the PL cortex and NAc shell. Tissue was homogenized in a RIPA buffer (Thermo Fisher Scientific, IL) containing protease inhibitors (Roche Diagnostics, Indianapolis, IN), and centrifuged at 12,000 rpm at 4 °C for 20 min. The supernatant was separated and total protein was measured using the Pierce BCA assay (ThermoScientific, Rockford, IL). For Western blot, approximately 10 μg of protein was loaded and separated on Bolt 4%–12% Bis–Tris mini gels (Thermo Fisher Scientific, IL), followed by transfer onto polyvinylidene difluoride membranes (Merck Millipore Ltd., Billerica, MA). Blots were cut at appropriate position into different pieces according to molecular sizes of target proteins. Then, blots were probed with rabbit anti-mGluR3 antibody (ab166608, 1:1500, Abcam, MA), rabbit anti-GLT-1 antibody (ab41621, 1:1500, Abcam, MA), or mouse anti-GAPDH antibody (G8795, 1:10,000; Sigma-Aldrich, CO) followed by incubation with appropriate IRDye^®^ secondary antibodies (1:15,000, LI-COR^®^, Lincoln, NE). Each blot was probed once and no stripping or re-probing was conducted. Blots were detected with an ODYSSEY^®^ CLx (LI-COR^®^, Lincoln, NE) fluorescent imaging system. The signal was quantified using Image Studio™ software (LI-COR^®^, Lincoln, NE, United States).

### Microdialysis

This procedure examined effects of ethanol or water drinking (*n* = 6–7/group) on basal extracellular glutamate concentrations in the PL cortex. P rats were used because our previous study demonstrated that chronic ethanol drinking enhanced basal extracellular glutamate concentrations in NAc shell and VTA in P rats (Ding et al., 2013). Briefly, female P rats were randomly divided into two groups (*n* = 6–7/group) with one group receiving 24 h continuous access to 15% ethanol vs. water in a two-bottle choice paradigm and the other group receiving water only for approximately 8 weeks. Shortly after the last drinking session, rats were implanted with a guide cannula aimed at PL cortex. After recovery for 2–3 days, microdialysis probes containing a 1.5 mm-long active membrane (I.D. × O.D = 200 μm × 216 μm, molecular weight cut-off: 13,000, Spectrum Laboratories, Inc., Rancho Dominguez, CA, United States) were inserted the PL cortex. Microdialysis was conducted ∼24 h later. No-net-flux microdialysis was conducted as previously described (Ding et al., 2013, 2017). Briefly, probes were connected to a Harvard pump via a PE20 tubing (I.D. = 0.38 mm; Becton Dickinson & Co., Franklin Lakes, NJ). Microdialysis started with a 90-min washout period during which aCSF (140.0 mM NaCl, 3.0 mM KCl, 1.2 mM CaCl_2_, 2.0 mM Na_2_HPO_4_.7H_2_O, 1.0 mM MgCl2, pH 7.2–7.4) was perfused through probes, followed by collection of 4–5 baseline samples. Then, four different concentrations of glutamate (1, 5, 10, or 20 μM in aCSF) were perfused through probes in a random order with each concentration perfused for 25 min. After all glutamate concentrations were perfused, the perfusion medium was changed to aCSF for an additional 20 min. Samples were collected every 5 min at a flow rate of 2.0 μl/min. Samples were frozen at −80 °C until glutamate analysis.

### HPLC

Glutamate content in microdialysis samples were analyzed using HPLC as previously described (Ding et al., 2013, 2017). Briefly, samples underwent precolumn derivatization with o-phthalaldehyde using an ESA Model 542 autosampler (ESA Inc., Chelmsford, MA). After derivatization, samples were injected onto a reversed-phase column (BDS Hypersil C18 Pioneer, 150 × 2 mm; Thermo Fisher Scientific, Waltham, MA) with a mobile phase consisting of 25% methanol (v/v) and 100 mM Na_2_HPO_4_.7H_2_O at pH 6.75. The mobile phase was delivered by an ESA 582 solvent delivery system. Glutamate was separated and detected with a BAS LC-4C amperometric detector. The oxidation potential was set at +550 mV and the sensitivity was set at 0.2 μA. The output was captured with the ChromPerfect chromatography data analysis system (Justice Laboratory Software, Denville, NJ). The concentration of glutamate was quantified by comparing peak area with an external standard curve.

### Microinjection

This study determined involvement of glutamate transporters in ethanol drinking. Wistar rats were selected because this strain of rats were used in our previous study that examined effects of manipulation of glutamate transmission on ethanol drinking (Ding et al., 2017). Briefly, male Wistar rats received free access to water and 20% ethanol in an intermittent access, two-bottle choice paradigm with ethanol available during three 24 h sessions each week (Monday, Wednesday, Friday). This is a validated procedure that promotes escalation of ethanol drinking in Wistar rats (Carnicella et al., 2014; Ding et al., 2017; Simms et al., 2008). After 14 weeks of drinking, rats were implanted with a guide cannula aimed at the lateral ventricle. Following at least 3 days of recovery, rats returned to intermittent ethanol drinking for ∼2 weeks to regain baseline drinking. Rats were then divided into two groups (*n* = 5/group) with one group receiving microinjection of LDN-212320 and the other group receiving vehicle treatment. During microinjection, a 28-gauge microinjector (Plastics One Inc, Roanoke, VA, United States) was inserted into the target region with 1 mm extension beyond the guide cannula. The microinjector was connected via a PE50 tubing to a 25-μl Hamilton syringe mounted on an infusion pump (Harvard Apparatus, Holliston, MA, United States). Vehicle or LDN-212320 (1.0 μg) was microinfused into the lateral ventricle in 1 μl over 1 min. After microinjection, the injector remained in place for one additional minute before removal. Each rat received 2 microinjections with one at ∼24 h and the other at ∼3 h prior to ethanol drinking. Rats started ethanol drinking as usual and continued for 2 weeks after the treatment. LDN-212320 is a translational activator of GLT-1 and has been shown to provide neuroprotection in animal models of pain and neurological diseases with excessive extracellular glutamate, including amyotrophic lateral sclerosis, epilepsy, and Alzheimer’s disease (Alotaibi and Rahman, 2019; Kong et al., 2014; Takahashi et al., 2015; Xing et al., 2011). This study was based on findings that several antibiotics reduced ethanol drinking in rats through enhancement of GLT-1 expression in the mesocorticolimbic system (Alhaddad et al., 2014; Goodwani et al., 2015; Qrunfleh et al., 2013; Sari et al., 2011).

### Histology

At the end of experiments, rats were euthanized with CO_2_ overdose (CO2 is delivered at flow rates for 30%–50% chamber volume per minute), and bromophenol blue was microinjected or perfused through microdialysis probes, and placements of microinjection sites and microdialysis probes were verified following procedures previously described (Ding et al., 2015a; Engleman et al., 2020). Rats with wrong placements were excluded from analysis.

### Statistical analysis

Data were expressed as Mean ± SEM. For time course data in ICSA and drinking experiments, repeated measures ANOVAs were employed followed by Bonferroni multiple comparisons. For Western blot, densitometric data for the protein of interest were first normalized against the loading control, i.e., GAPDH. Then, values from Wistar rats were averaged as 100% and were used to normalize values from P rats. The normalized data were analyzed with student *t*-tests. No-net-flux microdialysis data were analyzed as previously described (Ding et al., 2013, 2017). Briefly, glutamate concentrations perfused through probes and obtained from dialysis samples were designated as [Glu_*in*_] and [Glu_*out*_], respectively. The net gain or loss of glutamate was defined as [Glu_*in*_]–[Glu_*out*_] and was calculated for each time point. These [Glu_*in*_]–[Glu_*out*_] values were plotted as the “y” axis against the values of [Glu_*in*_] as the “x” axis to construct regression lines. The zero net gain or loss, i.e., [Glu_*in*_]–[Glu_*out*_] = 0, corresponds to the no-net-flux point. The x intercept of the regression line at the no-net-flux point corresponds to extracellular glutamate concentration. The slope of the regression line is defined as extraction fraction [Ed], which is an index of glutamate uptake. The slope and x-intercept were determined with linear regression for each rat, and group comparisons were analyzed with student *t*-tests. Significance level was set at *p* < 0.05.

## Results

### Effects of the mGluR2/3 agonist LY379268 on ethanol ICSA

**FIGURE 1:**
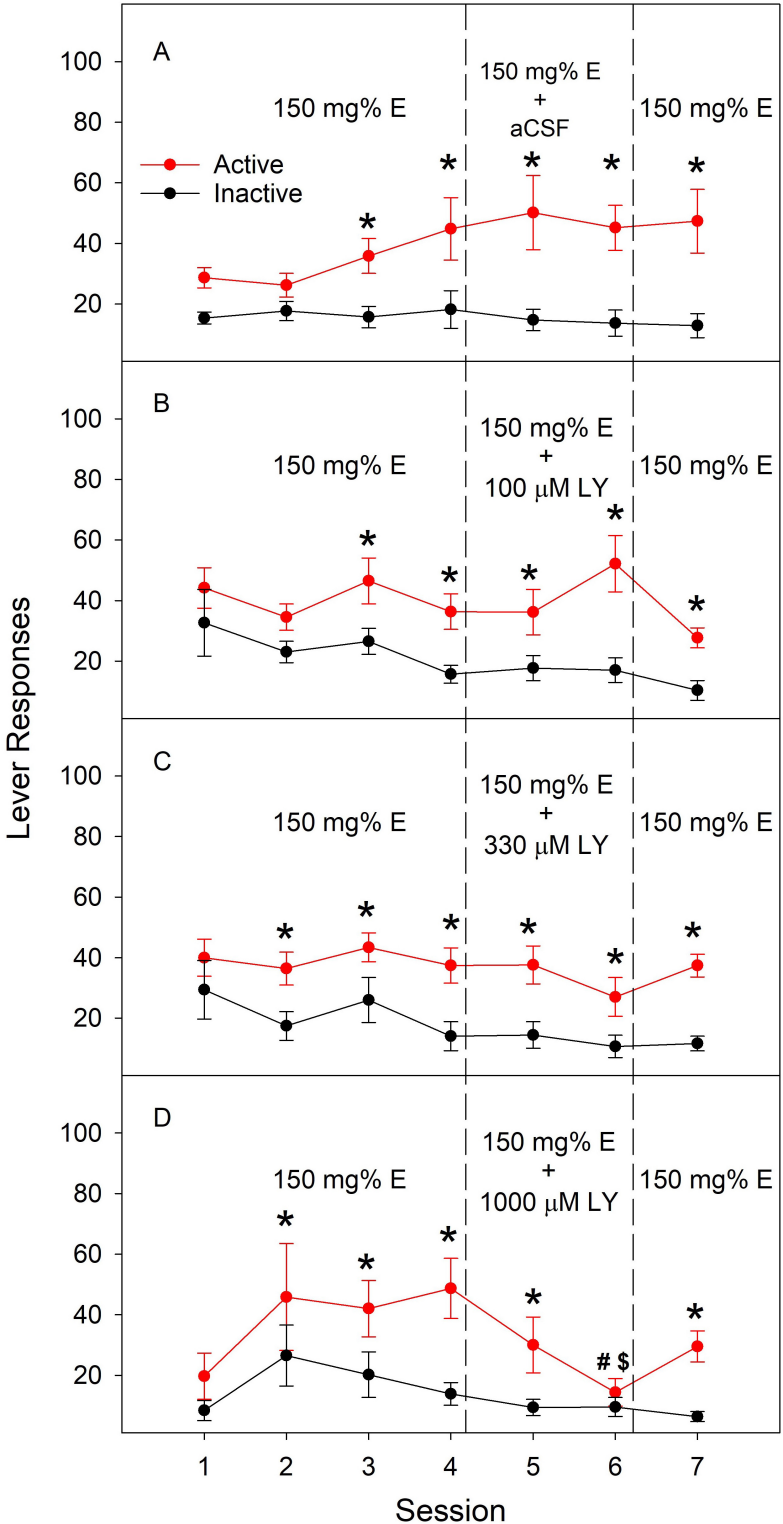
**(A)** Effects of aCSF; **(B)** effects of 100 μM LY; **(C)** effects of 330 μM LY; **(D)** effects of 1000 μM LY. Effects of co-infusion of the metabotropic glutamate receptor 2/3 agonist LY379268 (LY) or artificial cerebrospinal fluid (aCSF) with 150 mg% ethanol (E) into the prelimbic cortex on lever responses during intracranial self-administration in female Wistar rats. Repeated measures ANOVA: session × treatment × lever: F_18_, _114_ = 2.0, *p* < 0.05. *Active lever responses significantly greater than inactive lever responses during the same session. ^#^Active lever responses significantly lower than those in the vehicle group and the 100 μM LY group during session 6. ^$^Active lever responses significantly lower than those during the last acquisition session, i.e., session 4.

Figure 1 depicts effects of co-infusion of LY379268 on ethanol ICSA into the PL cortex. Rats gradually developed lever differentiation during acquisition with more active responses than inactive responses in all groups. LY379268 at 100 μM (Figure 1B) or 300 μM (Figure 1C) did not significantly alter active responses or lever differentiation. LY379268 at 1,000 μM reduced active responses and eliminated lever differentiation during the second co-infusion session (Figure 1D). Active responses during session 6 with 1,000 μM LY379268 were significantly lower than those during the 4*^th^* acquisition session (Figure 1D), as well as those in the vehicle (Figure 1A) and LY379268 100 μM (Figure 1B) groups during session six. Removal of LY379268 from ethanol solution returned active responses toward acquisition levels (Figure 1D). These results indicate that activation of mGluR2/3 dose-dependently attenuated ethanol ICSA into the PL cortex.

**FIGURE 2:**
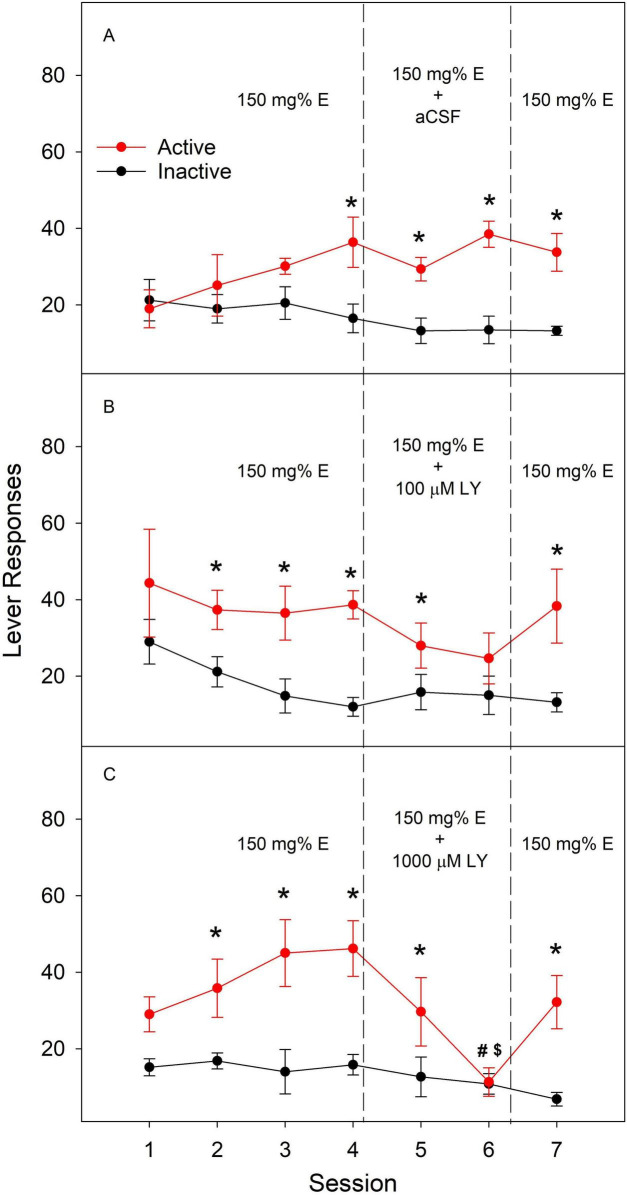
**(A)** Effects of aCSF; **(B)** effects of 100 μM LY; **(C)** effects of 1000 μM LY. Effects of co-infusion of the metabotropic glutamate receptor 2/3 (mGluR2/3) agonist LY379268 (LY) or artificial cerebrospinal fluid (aCSF) with 150 mg% ethanol (E) into the nucleus accumbens shell on lever responses during intracranial self-administration in female Wistar rats. Repeated measures ANOVA: session × lever × treatment: F_12_, _102_ = 2.3, *p* = 0.014. *Active lever responses significantly greater than inactive lever responses. ^#^Active lever responses significantly lower than those in the vehicle group during the same session. ^$^Active lever responses significantly lower than those during the last acquisition session, i.e., session 4.

Figure 2 depicts effects of LY379268 co-infusion on ethanol ICSA into the NAc shell. During acquisition, rats gradually developed lever differentiation and obtained more active responses than inactive responses in all groups. LY379268 at 100 μM did not alter active lever responses during either co-infusion session, although it eliminated lever differentiation during the second co-infusion sessions (Figure 2B). LY 379268 at 1,000 μM significantly reduced active lever responses during session six compared to the vehicle treatment (Figure 2A) and active responses during the 4*^th^* acquisition session (Figure 2C). Removal of LY379268 from ethanol solution returned active responses toward acquisition baseline (Figure 2C). These results indicate that activation of mGluR2/3 attenuated ethanol ICSA into the NAc shell.

### Protein levels of mGluR3 and GLT-1 in the PL cortex and NAc shell between P and wistar rats

**FIGURE 3:**
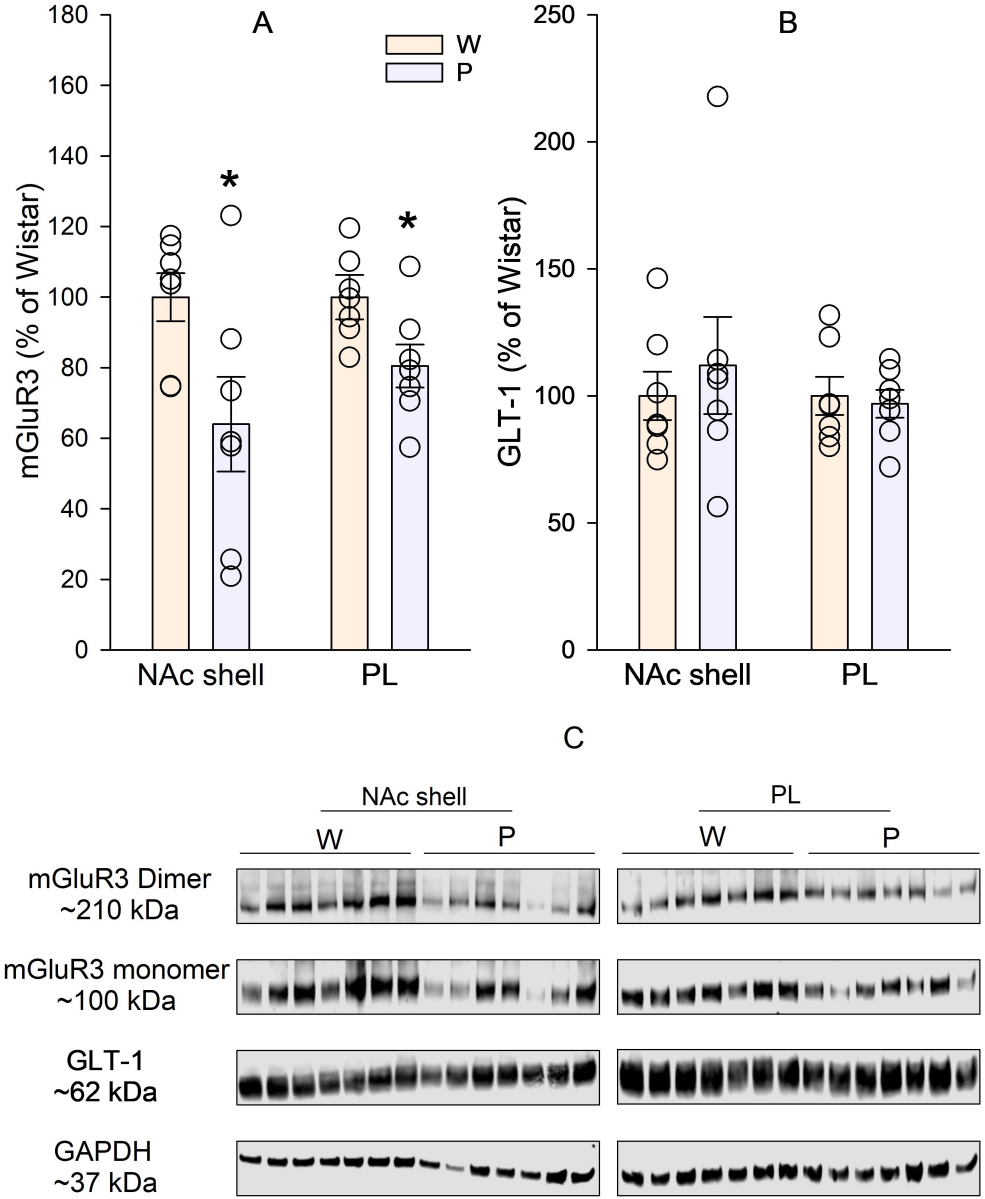
Basal protein levels of the metabotropic glutamate receptor 3 (mGluR3) and glutamate transporter 1 (GLT-1) in the prelimbic (PL) cortex and nucleus accumbens (NAc) shell in ethanol-naïve male Wistar (W) and alcohol-preferring P rats. **(A)** mGluR3 levels. PL: *t*_12_ = 2.5, *p* < 0.05; NAc shell: *t*_12_ = 2.4, *p* < 0.05. **(B)** GLT-1 levels. NAc shell: *t*_12_ = 0.6, *p* > 0.05; PL: *t*_12_ = 0.4, *p* > 0.05. **(C)** Western blot image of mGluR3, GLT-1, and GAPDH. *Significantly lower in P rats than in W rats.

Basal protein levels of mGluR3 were significantly lower in both NAc shell and PL cortex in P rats than in Wistar rats (Figures 3A, C). On the other hand, protein levels of GLT1 were not significantly different in either NAc shell or PL cortex between P and Wistar rats (Figures 3B, C).

### Effects of chronic ethanol drinking on basal extracellular glutamate transmission within the PL cortex

**FIGURE 4:**
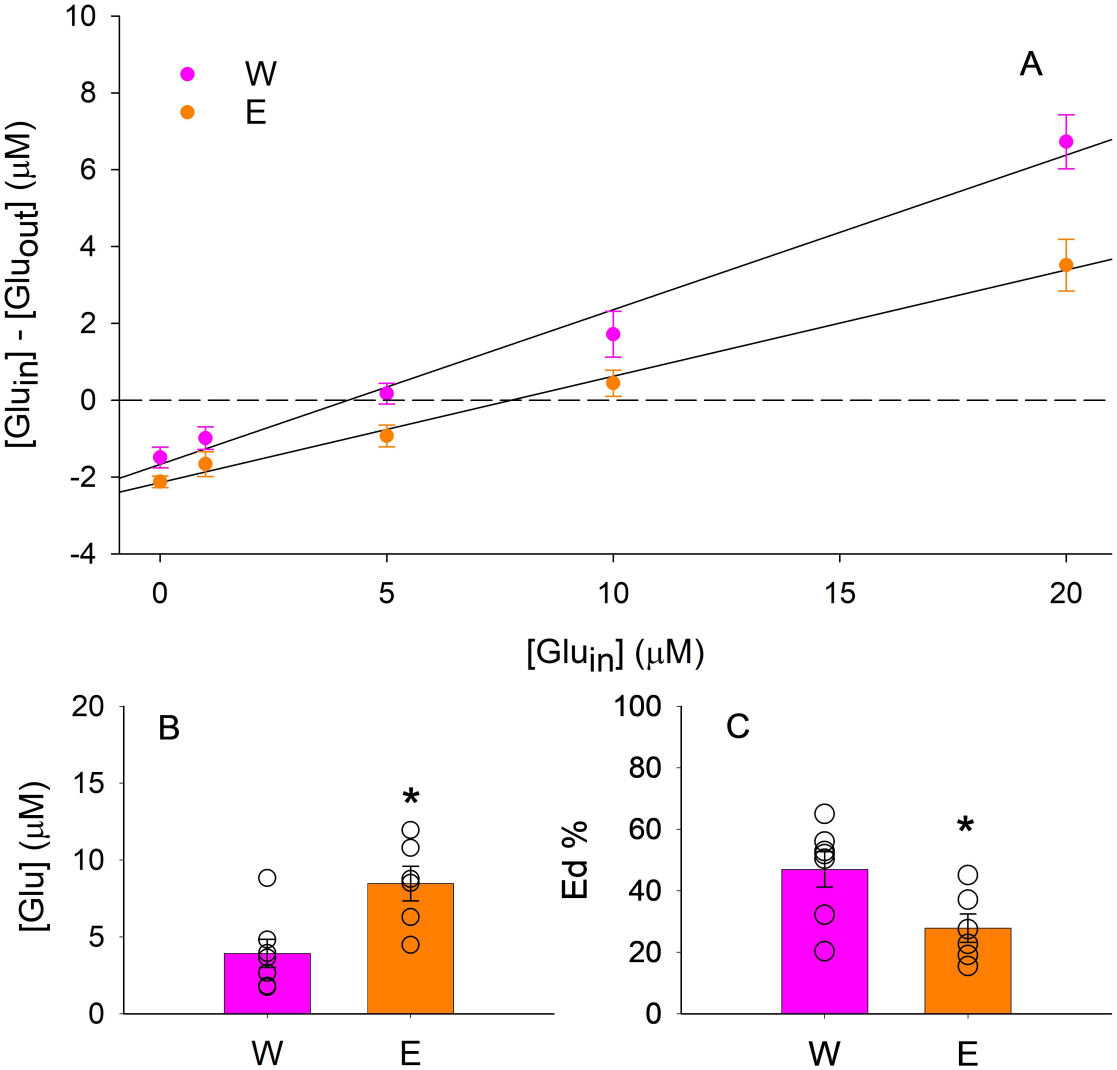
Effects of chronic ethanol (E) or water (W) drinking on basal extracellular glutamate concentrations and clearance within the prelimbic cortex in female alcohol-preferring P rats. **(A)** Linear regression plots of glutamate. [Glu_*in*_]: glutamate concentrations perfused through probes. [Glu_*out*_]: glutamate concentrations obtained from dialysis samples. [Glu_*in*_]–[Glu_*out*_]: net gain or loss of glutamate for each sample. [Glu_*in*_]–[Glu_*out*_] = 0 corresponds to the no-net-flux points. **(B)** Basal extracellular glutamate concentrations higher in the ethanol group than those in the water group (*t*_11_ = 3.1, *p* < 0.05). **(C)** Extraction fraction (Ed) values of glutamate, an index of glutamate uptake, lower in the ethanol group than those in the water group (*t*_11_ = 2.5, *p* < 0.05). *Significantly different between E and water groups.

P rats in the ethanol group consumed an average of 4.9 ± 0.5 g/kg/day ethanol during the last 3 weeks prior to surgery. Basal extracellular glutamate concentrations were significantly greater in the ethanol group than those in the water group (Figures 4A, B). Eds were significantly lower in the ethanol group than those in the water group (Figures 4A, C). These results suggest that ethanol drinking increased basal extracellular glutamate levels and reduced glutamate uptake.

### Effects of LDN-212320 on ethanol drinking

**FIGURE 5:**
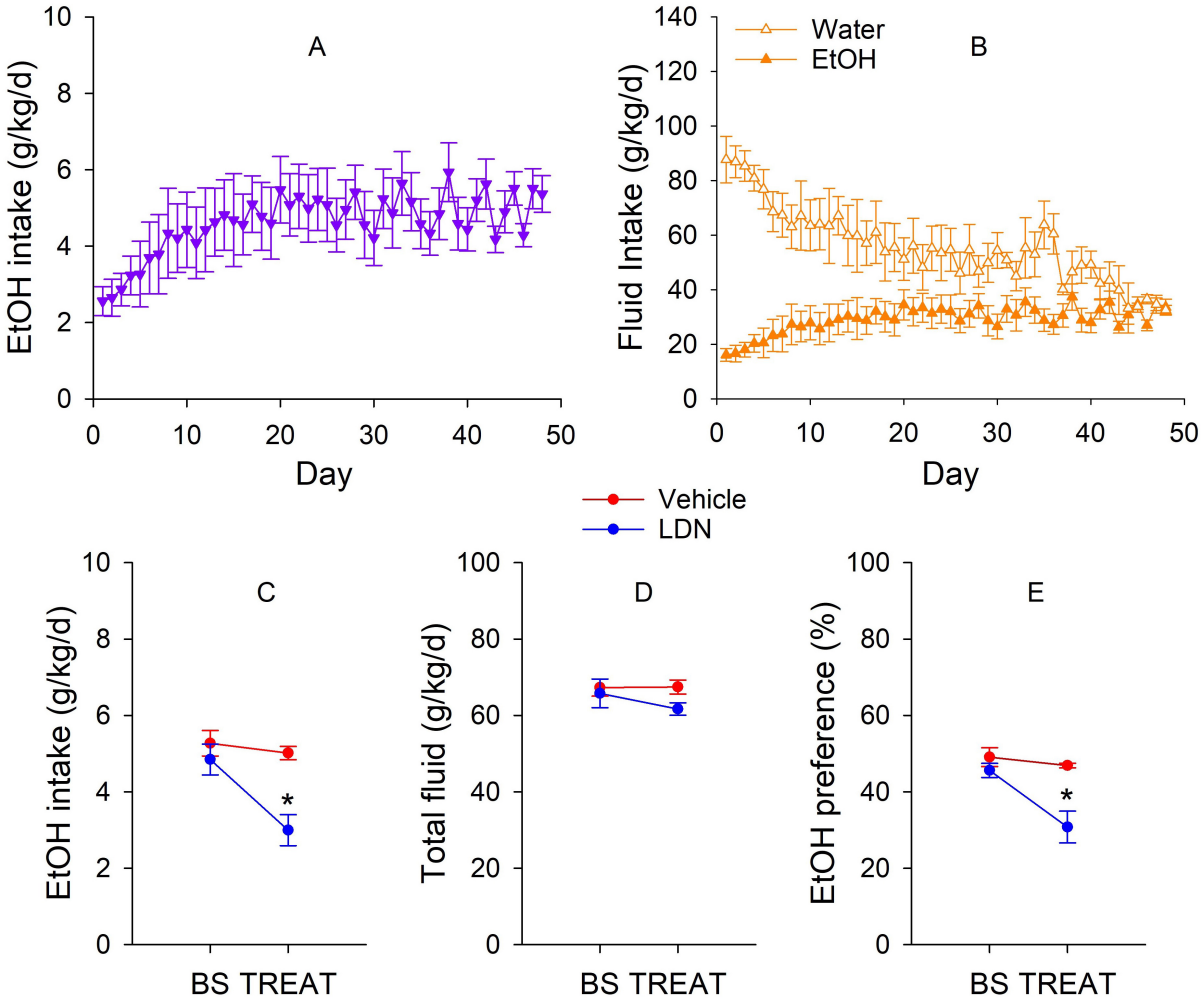
Effects of intraventricular microinjection of LDN-212320 or vehicle on ethanol (EtOH) drinking in male Wistar rats. **(A)** Escalation of EtOH during intermittent access over days (F_47_, _423_ = 1.9, *p* < 0.001). **(B)** Gradual increase of EtOH fluid intake (F_47_, _423_ = 4.4, *p* < 0.001) and gradual decrease of water fluid intake (F_47_, _423_ = 1.5, *p* = 0.019) during intermittent access over days. **(C)** LDN reduced EtOH intake. Repeated measures ANOVA: day × treatment: F_1_, _8_ = 9.3, *p* < 0.01. **(D)** LDN had no effect on total fluid consumption. Repeated measures ANOVA: day x treatment: F_1_, _8_ = 1.2, *p* = 0.30. **(E)**: LDN reduced EtOH preference. Repeated measures ANOVA: day × treatment: F_1_, _8_ = 8.8, *p* < 0.05. BS: baseline levels averaged from the last three drinking sessions. *Significantly lower than BS and the vehicle treatment.

Ethanol intake and fluid intake prior to treatment are shown in Figures 5A, B. Wistar rats (*n* = 10 in total) gradually escalated ethanol intake from ∼2.5 g/kg/d during the first week to ∼5 g/kg/d by the end of the 6*^th^* week, and remained at this level during the remainder of drinking (Figure 5A). Ethanol fluid intake gradually increased whereas water fluid intake gradually decreased (Figure 5B). Effects of LDN-212320 or vehicle (*n* = 5/group) on ethanol drinking are shown in Figures 5C–E. LDN-212320 significantly reduced ethanol intake (Figure 5C) and preference (Figure 5E) compared to corresponding baseline levels and those in the vehicle group. LDN-212320 did not alter total fluid consumption (Figure 5D). Two weeks after treatment, ethanol intake returned to baseline levels and was not different between the LDN-treated and vehicle-treated groups (5.4 ± 0.7 vs. 5.2 ± 0.6 g/kg/day, respectively; *t*_8_ = 0.2, *p* = 0.8). These results indicate that LDN-212320 reduced ethanol drinking in a reversible manner.

## Discussion

The current study demonstrates that (a) co-infusion of the mGluR2/3 agonist LY379268 with ethanol attenuated ethanol ICSA into the PL cortex and NAc shell, (b) alcohol preferring P rats displayed lower levels of mGluR3 protein, but not GLT-1, in the PL cortex and NAc shell compared to Wistar rats, (c) chronic ethanol drinking increased basal extracellular glutamate concentrations and reduced extracellular glutamate clearance within the PL cortex, and (d) intraventricular microinjection of LDN-212320, a GLT-1 enhancer, decreased ethanol drinking. Taken together, these results suggest that glutamate transmission with the PL cortex and NAc shell play important roles in ethanol reinforcement and drinking.

Both NAc shell and PL cortex are important anatomical substrates supporting the reinforcing effects of ethanol (Engleman et al., 2009, 2020). Our data indicate that mGluR2/3-mediated glutamate transmission regulates the reinforcing effects of ethanol within these brain regions (Figures 1, 2). These results add to our previous findings demonstrating the importance of D_2_ receptor-mediated dopamine transmission in ethanol ICSA in PL cortex (Engleman et al., 2020), and GABA_A_ receptor-mediated GABA transmission and 5-HT_3_ receptor-mediated 5-HT transmission in ethanol ICSA in NAc shell (Ding et al., 2015a). Our results are consistent with previous findings that administration of mGluR2/3 agonists or a positive allosteric modulator either systemically or locally into discrete brain regions, including NAc, reduces voluntary alcohol drinking, ethanol self-administration, and relapse to ethanol seeking (Augier et al., 2016; Bäckström and Hyytiä, 2005; Besheer et al., 2010; Griffin et al., 2014; Sidhpura et al., 2010). As a mGluR2/3 agonist, LY379268 reduces extracellular glutamate levels in the NAc shell (Griffin et al., 2014). In addition, intra-NAc LY379268 has been shown to decrease local extracellular dopamine levels (Greenslade and Mitchell, 2004). These results suggest that LY379268 reduces glutamate release, which may subsequently lead to a reduction in extracellular dopamine levels. It is possible that this impaired dopamine transmission may contribute to decreasing ethanol reinforcement, at least in the NAc. Besides their main pre-synaptic location, mGluR2/3 are also found at postsynaptic sites and on glial cells (Petralia et al., 1996). Potential contribution of these receptors to effects of LY379268 remains to be determined.

LY379268 is a potent agonist of both mGluR2 and mGluR3 with EC50 values in low nM range (Imre, 2007). Therefore, the contribution from each individual receptor remains unclear. One limitation of our study is that the exact concentrations of LY379268 at tissue surrounding the microinjection site remain unknown mainly due to the highly dynamic process involved in rapid drug diffusion from the injection site. LY379268 has been shown to interact with dopamine D_2_ receptors with high affinity and Ki values comparable to those for mGluR2/3 (Seeman and Guan, 2008) but also see (Zysk et al., 2011). Therefore, a potential involvement of D_2_ receptors may not be excluded. One notable side effect of LY379268 is its inhibition of locomotor activity at high concentrations (Imre, 2007). For example, microinjection of LY379268 at 1.7 mM into the NAc reduced locomotor activity in rats (Besheer et al., 2010). This concentration is higher than those used in our study. LY379268 at 1 mM in the NAc did not alter ethanol self-administration in rats (Besheer et al., 2010). LY379268 up to 2 mM in the NAc failed to reduce voluntary ethanol drinking in either dependent or non-dependent mice (Griffin et al., 2014). These results suggest that LY379268 at 1 mM used in our study may not lead to significant locomotor impairment. In addition, LY379268 did not alter inactive responses in our study. Taken together, although potential LY379268-induced locomotor impairment may not be excluded, such likelihood may be low. In addition, another limitation of the current ICSA studies is potential tissue damage due to repeated ICSA sessions. Data from the current and previous studies (Ding et al., 2009b,2015a, b; Engleman et al., 2009, 2020) indicate that rats typically maintain high responses after 7–8 repeated ICSA sessions comparable to those during acquisition, suggesting that tissue damage may not significantly alter ICSA behavior.

Local effects of ethanol on glutamate transmission in both PL cortex and NAc shell remain largely unknown. Our data suggest that ethanol infusion into these sub-regions may possibly increase local extracellular glutamate levels to facilitate ethanol ICSA. This hypothesis is consistent with findings from microdialysis studies that acute intraperitoneal injection of ethanol increases extracellular glutamate levels in the NAc in rats at doses including 0.5, 1.0, and 2.0 g/kg (Dahchour et al., 2000; Moghaddam and Bolinao, 1994; Quertemont et al., 2002; Selim and Bradberry, 1996). On the other hand, several studies show that systemic injection of ethanol at doses above 2 g/kg decreases extracellular glutamate levels in the NAc (Moghaddam and Bolinao, 1994; Quertemont et al., 2002). For the PL cortex, microdialysis studies have found inconsistent results. One study shows that intra-gastric administration of ethanol at 2 g/kg increases extracellular glutamate levels in the mPFC of mice (Hwa et al., 2015), whereas another reports that intraperitoneal injection of ethanol at doses up to 2 g/kg did not alter extracellular glutamate levels in the mPFC in rats (Selim and Bradberry, 1996). Early *in vitro* studies indicate that ethanol incubation increases glutamate release from cerebral cortex culture or brain slices containing frontal cortex (McBride et al., 1986; Singh et al., 1994). Acute ethanol injection increases tissue content of glutamate in the cerebral cortex (Ameeta Rani et al., 1985). It should be noted that systemic administration of ethanol in these studies most likely have broad effects besides the NAc shell or PL cortex, thus lacking anatomical specificity. Therefore, the localized effects of ethanol on glutamate levels within the PL cortex and NAc shell remain to be determined.

Our results indicate that strain differences exist between alcohol-preferring P and Wistar rats in basal levels of mGluR3 protein with lower levels in P than Wistar rats in both PL cortex and NAc shell. Our previous study indicate that P rats exhibit greatly reduced mGluR2 protein expression in both NAc shell and PL cortex compared to Wistar rats (Ding et al., 2017). Together, these data suggest that impairment in both mGluR2 and mGluR3 expression may be associated with high alcohol preference and consumption in P rats. These results are consistent with genomic and microarray studies that demonstrate excessive differences in glutamate system between rat models with bidirectional selection for alcohol preference (Lo et al., 2016; McBride et al., 2012, 2013). Our findings are consistent with a recent study showing that significant impairment in mGluR2/3 function in the PL cortex is associated with ‘alcohol use disorder’-related behaviors in rats (Domi et al., 2024). However, such an association was not found in NAc in the Domi et al. (2024) study, which is different from our study. Taken together, these studies suggest that the mGluR2/3 in the PL cortex may be more consistently involved in alcohol addiction. However, it should be noted that a previous study from our lab shows that reduction of mGluR2 expression via virus-mediated shRNA in the PL cortex did not alter alcohol drinking in rats (Ding et al., 2017). Although these negative results may be related to shRNA-mediated partial reduction of mGluR2 (∼60%–70% reduction in mRNA expression and ∼40% reduction in protein expression), the causal role of mGluR2/3 deficit from discrete brain regions in predisposition to high alcohol preference, consumption, and addiction is more complicated than expected, and warrants more research.

Since mGluR2/3 are mainly glutamate auto-receptors providing important feed-back regulation of glutamate release, these results suggest that basal extracellular glutamate levels may be different between P and Wistar rats. A previous study using the quantitative no-net-flux microdialysis technique demonstrates that basal extracellular glutamate concentrations are lower in P rats than Wistar rats in both PL cortex and NAc shell (Ding et al., 2017). These results are sharply opposite to the prediction based on the assumption that the deficit in mGluR2/3 auto-receptors would result in disinhibition of glutamate release and subsequent elevation of extracellular glutamate levels. This apparent paradox suggests that other mechanisms may contribute to lower extracellular glutamate levels, e.g., excessive glutamate clearance. GLT-1 is the predominant glutamate transporter that is responsible for more than 90% glutamate uptake (Danbolt, 2001). However, our results show that GLT-1 protein levels are not different in PL cortex or NAc shell, suggesting similar glutamate uptake between P and Wistar rats. Given these, it is possible that glutamate release may be significantly diminished in P than Wistar rats to account for lower extracellular glutamate levels in P rats than Wistar rats. This hypothesis should be further tested in future studies.

Voluntary ethanol drinking increases basal extracellular glutamate concentrations within the PL cortex. This is consistent with previous studies demonstrating that chronic ethanol drinking or vapor exposure elevates basal glutamate levels in the mPFC in mice and rats (Hermann et al., 2012; Hwa et al., 2015). In addition, our previous no-net-flux microdialysis study demonstrate that chronic ethanol drinking also enhances basal extracellular glutamate levels in the NAc shell (Ding et al., 2013). These results suggest that chronic ethanol drinking induces a hyper-glutamatergic state characterized by elevated basal extracellular glutamate levels within the PL cortex and NAc shell, which may play a role in the maintenance of ethanol drinking. It should be noted that the current microdialysis was conducted ∼3 days after the last drinking session. Both the Ding et al., 2013, Hwa et al., 2015 studies indicate that glutamate levels started to increase during the acute (∼6–8 h) withdrawal. The Hermann et al. (2012) study indicate that glutamate levels increased at both 12 and 60 h after ethanol cessation. Taken together, our results suggest that chronic ethanol drinking may lead to relatively persistent elevation of basal extracellular glutamate concentrations in the PL cortex, at least for 3 days after ethanol cessation.

The no-net-flux analysis also reveals a decrease in extraction fraction in the PL cortex following ethanol drinking, suggesting that ethanol exposure may impair glutamate clearance, which may contribute, at least in part, to the ethanol-induced elevation of basal extracellular glutamate concentrations. A previous study indicates that prolonged ethanol drinking impairs glutamate uptake activity in an *ex vivo* assay and down-regulates glutamate transporter binding sites in cerebral cortex in rats, suggesting ethanol drinking may reduce both protein expression and function of glutamate transporters (Schreiber and Freund, 2000). Similar ethanol-induced impairment of glutamate transporter expression and glutamate uptake has been reported in the NAc (Ding et al., 2013; Kapasova and Szumlinski, 2008; Melendez et al., 2005; Sari et al., 2013b). In addition, our previous study suggests that chronic ethanol drinking may down-regulate mGluR2/3 function in the brain of P rats, which may lead to dis-inhibition of synaptic glutamate release and elevation of extracellular glutamate levels (Ding et al., 2016). Since no-net-flux microdialysis is not designed to determine glutamate release, it may not be excluded that mGluR2/3 down-regulation may contribute to ethanol-enhanced basal extracellular glutamate concentrations.

The up-regulation of extracellular glutamate transmission within the PL cortex and NAc shell may play a critical role in ethanol drinking. Our results demonstrate that intraventricular microinjection of LDN-212320 suppressed ethanol intake and preference without altering total fluid consumption. LDN-212320 is a selective and potent GLT-1 activator eliciting time-dependent elevation of GLT-1 with GLT-1 levels increasing at ∼2–3 h, peaking at ∼24 h, then returning during the next 48 h (Kong et al., 2014; Xing et al., 2011). In our study, the first LDN-212320 microinjection at ∼24 h before ethanol drinking was intended to maximize GLT-1 enhancement at the beginning of ethanol session. The second microinjection at ∼3 h prior to ethanol drinking was intended to maintain high levels of GLT-1 elevation throughout the 24 h drinking session. In addition, ethanol intake returned to baseline levels after 2 weeks following the LDN-212320 treatment, suggesting that LDN-induced ethanol reduction is reversible. This is consistent with the findings that GLT-1 returns to pre-treatment levels at ∼72 h after LDN treatment (Kong et al., 2014). Our findings suggest that LDN may up-regulate GLT-1 expression to inhibit ethanol drinking. It should be noted, though, that intra-ventricular administration of LDN is expected to have broad effects and the contribution from other brain regions may not be excluded.

These results agree with a substantial body of research demonstrating that a number of GLT-1 enhancers, represented by the beta-lactam antibiotic ceftriaxone, reduce ethanol drinking in rats (Alhaddad et al., 2014; Goodwani et al., 2015; Sari et al., 2013a). Conversely, previous studies demonstrate that intra-NAc administration of TBOA, a glutamate transporter inhibitor, increased ethanol drinking in mice (Griffin et al., 2014; Kapasova and Szumlinski, 2008). Taken together, these findings suggest that bi-directional manipulations of GLT-1 in the NAc may differentially regulate ethanol drinking.

Collectively, the results of the current study indicate important roles of glutamate transmission within the PL cortex and NAc shell in mediating acute reinforcing effects of ethanol, ethanol preference, and chronic ethanol drinking. One weakness of the current study is that only one sex and one strain of rats were included in each experiment. Such selection was mainly based on our previous studies involving similar procedures, which may allow comparisons between studies. However, this weakness limits the generalization of current findings to other sex and strains. Nonetheless, our data still enrich the existing literature implicating the role of glutamate transmission in the development of alcohol use disorder. This enhanced understanding may facilitate future development of effective therapeutic strategy combating alcohol use disorder.

## Data Availability

The raw data supporting the conclusions of this article will be made available by the authors, without undue reservation.
